# Computational and cellular exploration of the protein-protein interaction between *Vibrio fischeri* STAS domain protein SypA and serine kinase SypE

**DOI:** 10.1080/19420889.2023.2203626

**Published:** 2023-04-20

**Authors:** Morgan E. Milton, Karen L. Visick

**Affiliations:** a Department of Biochemistry and Molecular Biology, Brody School of Medicine, East Carolina University, Greenville, NC, USA; b Department of Microbiology and Immunology, Loyola University Chicago, Maywood, IL, USA

**Keywords:** Anti-sigma factor antagonists, biofilm, phosphorylation, protein-protein interaction, STAS, *Vibrio*

## Abstract

Anti-sigma factor antagonists SpoIIAA and RsbV from *Bacillus subtilis* are the archetypes for single-domain STAS proteins in bacteria. The structures and mechanisms of these proteins along with their cognate anti-sigma factors have been well studied. SpoIIAA and RsbV utilize a partner-switching mechanism to regulate gene expression through protein-protein interactions to control the activity of their downstream anti-sigma factor partners. The *Vibrio fischeri* STAS domain protein SypA is also proposed to employ a partner-switching mechanism with its partner SypE, a serine kinase/phosphatase that controls SypA’s phosphorylation state. However, this regulation appears opposite to the canonical pathway, with SypA being the more downstream component rather than SypE. Here we explore the commonalities and differences between SypA and the canonical single-domain STAS proteins SpoIIAA and RsbV. We use a combination of AlphaFold 2 structure predictions and computational modeling to investigate the SypA-SypE binding interface. We then test a subset of our predictions in *V.fischeri* by generating and expressing SypA variants. Our findings suggest that, while SypA shares many sequence and structural traits with anti-sigma factor antagonist STAS domain proteins, there are significant differences that may account for SypA’s distinct regulatory output.

## Introduction

**Figure 1.**
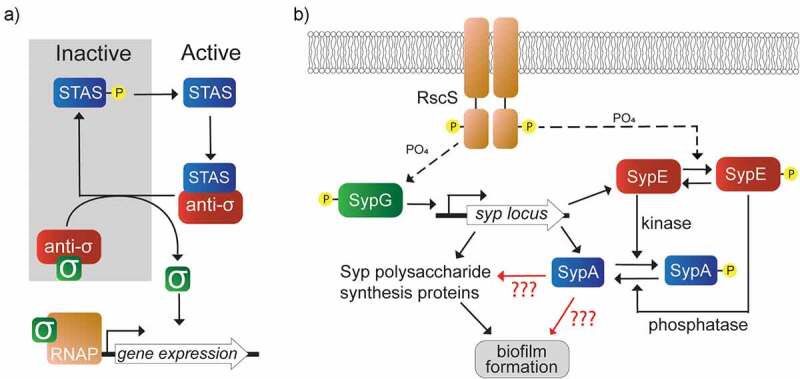
Comparison of regulatory pathways. a) the canonical pathway model for single-domain STAS proteins as anti-sigma factor antagonists. With no environmental trigger (grey region), the single-domain STAS protein (blue) is inactive. The anti-sigma factor (red) binds and sequesters its cognate sigma factor (green) preventing gene expression. An environmental signal leads to the dephosphorylation and activation of the STAS protein (white region). The STAS protein binds the anti-sigma factor, releasing the sigma factor and turning on gene expression. b) Model of the *syp*-dependent biofilm induction pathway in *V. fischeri*. Sensor kinase RscS (orange) indirectly activates SypG (green) which turns on *syp* transcription. RscS also regulates phosphorylation of SypE (red). Unphosphorylated SypE acts as a kinase that phosphorylates and inactivates SypA (blue), preventing biofilm formation.

Protein-protein interactions are often used to regulate protein activity. These interactions play diverse and central roles in all living organisms [1]. In bacteria, one common example of this phenomenon is the control of sigma factor activity by proteins known as anti-sigma factors [2]. The activity of the anti-sigma factors may be further governed by anti-sigma factor antagonists, also known as anti-anti-sigma factors. Such interactions have been termed “partner switching”, as the anti-sigma factor alternatively associates with either the sigma factor or the anti-sigma factor antagonist (Figure 1a) [3]. This series of protein-protein interactions plays an important role in bacterial gene regulation [4,5].

One class of anti-sigma factor antagonists consists of STAS (Sulfate Transporters and Anti-Sigma factor antagonist) domain proteins [2]. STAS domain proteins are encoded in most bacterial genomes and maintain a conserved structure with a high sequence divergence across proteins and species [3]. While some STAS domains are positioned within multi-domain sulfate transporters, as suggested by the name, others are single domain proteins that function through protein-protein interactions. For example, some single-domain STAS proteins interact within multi-protein complexes such as stressosomes [6,7]. The *Bacillus subtilis* STAS domain protein SpoIIAA is the first and perhaps the best characterized of the anti-sigma antagonist factor group of STAS proteins [8–12]. SpoIIAA binds to and sequesters the anti-sigma factor SpoIIAB. In turn, SpoIIAB phosphorylates SpoIIAA, facilitating its release. Once free, SpoIIAB can bind to and inhibit the activity of the sporulation-promoting sigma factor σ^F^. Inhibition of σ^F^ prevents sporulation until optimal conditions are reached. In the forespore, SpoIIAA is dephosphorylated by a phosphatase, restoring SpoIIAA sequestration activity, and allowing SpoIIAA to promote sporulation [13,14]. A similar scheme controls the activity of *B. subtilis* σ^B^. The phosphorylation status of the STAS anti-sigma factor antagonist RsbV is controlled by its target, the anti-sigma factor RsbW [15,16]. For both the SpoIIAA and RsbV pathways, the STAS domain protein acts upstream of its cognate anti-sigma factor serine kinase to control gene expression.

However, not all single-domain STAS proteins function like these well-understood models. In several more recently studied systems, the sense of the pathway is backwards and/or the STAS protein doesn’t function as an anti-sigma factor antagonists [2]. For example, phosphorylation of the *Bordetella bronchiseptica* STAS protein BtrV promotes binding rather than the canonical dissociation from its anti-sigma factor BtrW [17]. The *Rhodobacter capsulatis* STAS protein RbaV appears to be the downstream regulator of gene expression [18]. Furthermore, HmpV from *Nostoc punctiforme* is suggested to bind an unknown downstream target to regulate gene expression based on its phosphorylation state as governed by its anti-sigma factor [19]. These departures from the paradigm, combined with the pervasiveness of STAS domain proteins encoded within bacterial genomes, underscore the importance of understanding the diversity of mechanisms by which STAS proteins function.

Another example of a non-canonical single-domain STAS protein is SypA from *Vibrio fischeri*. SypA controls biofilm formation at an unknown level below transcription [20]. The activity of this small (105 aa) protein is governed by its phosphorylation state on Ser56. When unphosphorylated, SypA promotes biofilm formation and host colonization by *V. fischeri* [20]. The SypA phosphorylation state is controlled by SypE, a tripartite response regulator protein with a unique domain architecture [21]. SypE is composed of an N-terminal serine kinase domain, a central receiver (REC) domain, and a C-terminal serine phosphatase domain. While the canonical pathway would position SypA upstream to control the activity of SypE, this is not the direction of the regulation (Figure 1b) [20]. A double *sypA sypE* deletion mutant exhibits the biofilm-defective phenotype of the single *sypA* deletion mutant. This indicates that, of the two proteins, SypA functions as the downstream partner and has activity even in the absence of SypE.

SypA and SypE are part of a larger regulatory pathway involving the *syp* locus (Figure 1b). The *syp* locus encodes proteins necessary to synthesize and regulate a polysaccharide critical for biofilm formation and host colonization [22–24]. In this regulatory pathway, the sensor kinase RscS functions upstream of SypE and a second response regulator, SypG [25]. Activation of SypG via RscS induces transcription of the *syp* locus. When SypE becomes activated by RscS through a phosphotransfer reaction, SypE switches from a kinase to a phosphatase state [26]. This switch shifts SypE from being an inhibitory kinase that phosphorylates and inactivates SypA to a biofilm-promoting phosphatase [20].

This pathway model was elucidated using the overexpression of positive biofilm-inducing regulators, RscS and SypG. When *rscS* is overexpressed from a multicopy plasmid, *V. fischeri* produces a variety of biofilm phenotypes not normally made by the wild-type strain under standard laboratory conditions. These phenotypes include wrinkled colony formation, which is eliminated upon disruption of *sypA* or *sypG*, but not *sypE* [20,23,24,26]. In contrast, when *sypG* is overexpressed from a multicopy plasmid, no biofilm is produced [27]. This holds true even if a constitutively active allele of *sypG* is used, despite a substantial increase in transcription of the *syp* genes [25]. The situation changes when *sypG* is overexpressed in a *sypE* deletion mutant: now wrinkled colonies and other *syp*-dependent phenotypes are readily produced [27,28]. The model presented in Figure 1b is further supported by the expression of a non-phosphorylatable SypA variant (SypA-S56A). SypA-S56A also permitted the *sypG* overexpression strain to produce wrinkled colonies despite the presence of SypE [27]. Together, these data support the conclusion that SypA is the more downstream component, with SypE acting to control SypA’s activity but not vice versa. This conclusion implies that SypA exerts its activity via another, as yet unknown protein target.

The *syp* locus is largely conserved in most *Vibrio* species, including pathogens such as *V. parahaemolyticus* and *V. vulnificus* (where it is termed *rbd*), with a notable exception being *V. cholerae* [22,23,29]. In particular, *sypA* appears to be present in most sequenced *syp* loci. This conservation suggests that knowledge gleaned using the more characterized *V. fischeri* system may provide insights into pathogen function.

Because SypA is part of a well-characterized pathway but functions in a manner distinct from canonical STAS-based anti-sigma factor/anti-sigma factor antagonist-type interactions, it provides an opportunity to expand our comprehension of STAS domain proteins. To build a foundation for understanding non-canonical STAS function, we use computational modeling to predict the commonality and diversity of structural features between SypA and the well-characterized canonical STAS proteins, *B. subtilis* proteins SpoIIAA and RsbV. We test a subset of our predictions by generating and expressing SypA variants. Our findings suggest that the SypA-SypE binding interface has some key differences from SpoIIAA and RsbV and that SypA may use its putative SypE interface to also interact with an unknown partner to promote biofilm formation.

## Methods

### Computational modeling

The AlphaFold model of SypA was retrieved from the AlphaFold database for Uniprot ID Q5DYQ6 [30]. The SypA RoseTTAfold model was generated using the Robetta web server [31]. A model of dimeric SypE bound to SypA was generated using ColabFold [32] with default parameters. The top ranked model was selected for further refinement with ICM Pro version 3.9-2e (MolSoft). The model was refined using Monte Carlo simulations for global energy optimization for all atoms in the model outlined in the ICM User’s Guide. MolProbity [33] was used to evaluate global and local model quality. Figures were generated in PyMol (The PyMOL Molecular Graphics System, Version 2.5.2 Schrödinger, LLC). AlphaFold pLDDT coloring was generated using a PyMol extension [34].

### Sequence alignments

A structure-based sequence alignment was generated in ICM Pro version 3.9-2e (MolSoft). Computational models of SypA were superimposed onto crystal structures for *B. subtilis* SpoIIAA (PDB ID 1AUZ and 1BUZ), *B. subtilis* RsbV (PDB ID 6M36 and 6M37), *G. stearothermophilus* SpoIIAA (PDB ID 1TIL and 1TID), and *L. sphaericus* (PDB ID 1H4Z). Sequences were extracted from the PDB files and aligned through the ICM Pro multiple sequence alignment tool.

### Simulated SypA mutagenesis

The globally optimized model of the SypA-SypE heterotrimer was used to evaluate the impact of SypA point mutations on the SypA-SypE protein-protein interaction. Simulations were performed using the ICM Pro version 3.9-2e (MolSoft). For each mutation, the model was subjected to 10 rounds of biased probability Monte Carlo simulations using the Mutation – Protein Binding protocol outlined in the ICM User’s Guide.

### Strains and media

*V. fischeri* strain ES114 [35] was the parent strain used for these studies. Derivatives were made as described below and are listed in Table 1. *Escherichia coli* strains were used for the purposes of conjugation and plasmid maintenance and included Tam1 λ*pir*, DH5α, and π3813 [36].

**Table 1.** V. fischeri strains used in this study.

Strain	Genotype^1^	Reference
ES114	Wild Type	[35]
KV10032	Δ*sypA*::FRT	This study
KV10149	Δ*sypA*::FRT IG:*sypA*-HA	This study
KV10163	Δ*sypE*::FRT	This study
KV10247	Δ*sypA*::FRT IG:*sypA*-S56A-HA	This study
KV10248	Δ*sypA*::FRT IG:*sypA*-D20A-HA	This study
KV10249	Δ*sypA*::FRT IG:*sypA*-F53A-HA	This study
KV10331	Δ*sypA*::FRT IG:*sypA*-S56D-HA	This study
KV10332	Δ*sypA*::FRT IG:*sypA*-S57A-HA	This study
KV10334	Δ*sypA*::FRT IG:*sypA*-D22A-HA	This study
KV10401	Δ*sypA*::FRT IG:*sypA*-D20A, D22A	This study

^1^IG indicates the placement of *sypA* in the intergenic (IG) region between *yeiR* and *glmS*; all *sypA* alleles are driven by the *sypA* promoter and potentially the promoter for the upstream linked Erm^R^ cassette.

*V. fischeri* was maintained in LBS (LB-salt) medium [37], which contains 1% tryptone, 0.5% yeast extract, 2% sodium chloride, and 50 mM Tris pH 7.5. In addition, as described below, biofilm experiments were performed using LBS medium. For strain construction, Tris Minimal Medium (TMM) was used and contained 100 mM Tris pH 7.5, 300 mM NaCl, 0.1% ammonium chloride, 10 mM *N*-acetylglucosamine, 50 mM MgSO_4_, 10 mM KCl, 10 mM CaCl_2_, 0.0058% K_2_HPO_4_, and 10 mM ferrous ammonium sulfate. *E. coli* was maintained on LB medium, which contained 1% tryptone, 0.5% yeast extract, and 1% sodium chloride. Media were solidified as needed with agar to a final concentration of 1.5%. *V. fischeri* strains were generally grown at 28°C, except as described below for some biofilm experiments (for which 24°C was used), while *E. coli* strains were grown at 37°C. During strain construction, for biofilm experiments that relied on *sypG* or *rscS* overexpression, and in other contexts as needed, antibiotics were added to the medium to the following final concentrations: Ampicillin, 100 μg/mL, Chloramphenicol (Cm), 1 μg/mL (selection for chromosomal markers), 5 μg/mL (plasmid maintenance in *V. fischeri*), or 12.5 μg/mL (plasmid maintenance in *E. coli*), Erythromycin (Erm), 5 μg/mL, and Kanamycin (Kan), 50 μg/mL (for *E. coli*) or 100 μg/mL (for *V. fischeri*).

### Strain construction

Strains were constructed as previously described [38,39] using plasmids and primers listed in Tables 2 and 3, respectively. As needed for subsequent manipulations, plasmids (Table 2) were introduced into strains of interest using a tri-parental conjugation method with a strain carrying the helper plasmid pEVS104 as described previously [39,42]. For mutant construction (Δ*sypA*::FRT and Δ*sypE*::FRT), sequences (~500 bp) flanking the gene of interest were amplified using PCR with the high fidelity polymerase KOD (MilliporeSigma Novagen KOD DNA Polymerase). The upstream reverse and downstream forward primers contained linker sequences that were complementary, respectively, to forward and reverse primers used to amplify the Erm^R^ cassette from template pKV494 [38]. The three DNA fragments were fused together via Splicing by Overlap Extension PCR (“PCR SOEing”) [43] and amplified using outside primers. The resulting composite was used to transform a *V. fischeri* strain engineered to overproduce the competence regulator TfoX using plasmid plostfoX-Kan (Kan^R^) [41] and made competent by growth in TMM containing Kan. Following selection for the Erm^R^-marked deletion, the resulting colonies were purified and evaluated for the presence of the deletion via PCR, using the outside primers and either cell suspensions with Taq polymerase or genomic DNA with KOD. Finally, the Erm^R^ cassette that marked the deletion was removed by introducing plasmid pKV496, which expresses the Flp recombinase that recognizes and resolves the FRT sequences that flank the engineered Erm^R^ cassette. PCR was used to confirm that Erm^S^ colonies carried the unmarked deletion.

**Table 2.** Plasmids used in this study.

Plasmid	Description	Reference
pEAH73	*sypG*-overexpression plasmid, Cm^R^, Tet^R^	[25]
pJJC4	*tfoX, litR* O/E plasmid, Cm^R^	[40]
pKG11	*rscS*-overexpression plasmid, Cm^R^, Tet^R^	[23]
pKV494	pJET + FRT-Erm cassette, Ap^R^	[38]
pKV496	Flippase plasmid, Kan^R^	[38]
pKV502	pJET + *yeiR*, FRT-erm, and linker sequences, Ap^R^	[38]
pKV503	pJET + *glmS* and linker sequences, Ap^R^	[38]
plostfoX-Kan	*tfoX* overexpression plasmid, Kan^R^	[41]

**Table 3.** Primers used in this study.

Purpose	Primer #	Sequence^1^
Delete *sypA*	423	GGTTGACAGGTTTCTTGGCG
Delete *sypA*	1821	tcctgtgtgaTGAGCTGACTAATAAAAGTATTAG
Delete *sypA*	2808	taggcggccgcactaagtatggATTTGATTCGAATTGATGTAGTTC
Delete *sypA*	2809	ggataggcctagaaggccatggGTTAATAAAACAACGCATTAATTAG
Antibiotic cassette	2089	CCATACTTAGTGCGGCCGCCTA
Antibiotic cassette	2090	CCATGGCCTTCTAGGCCTATCC
Delete *sypE*	425	AGGGGTTCGTATTTCGTGACTC
Delete *sypE*	460	GCCTTGATAGGAGCATTATAATG
Delete *sypE*	2263	taggcggccgcactaagtatggATTCATGATTACACCACTGTTG
Delete *sypE*	2264	ggataggcctagaaggccatggCCCAATGACGATGCATTATTGC
*sypA* complementation	3083	ggataggcctagaaggccatggAGCTTCTTCCTTATAGTTATGATG
*sypA* complementation	3297	ttatgcataatctggaacatcatatggataATGCGTTGTTTTATTAACAGGAATTG
Complementation outside primer (*yeiR*)	2185	CTTGATTTATACAGCGAAGGAG
*tfox* complementation HA tag	2331	tatccatatgatgttccagattatgcataaCCATACTTAGTGCGGCCGCCTA
Complementation outside primer (*glmS*)	1487	GGTCGTGGGGAGTTTTATCC
*glmS* (nested)	2289	AATTGCTGTTGAAGCATCTCTG
Erm^R^ (internal)	2290	AAGAAACCGATACCGTTTACG
Erm^R^ (nested)	2291	CAGGTAAAGGGCATTTAACG
*sypA*-S56A mutation	4083	CTTTTTAGAT**g**CATCAGGTATTGGCGCTATTGTT
*sypA*-S56A mutation	4084	CAATACCTGATG**c**ATCTAAAAAGGCTACGTGTGATA
*sypA*-D20A mutation	4085	AGTTCAAGGTG**c**TATGGACGCCATCGGTTGTAG
*sypA*-D20A mutation	4086	TGGCGTCCATA**g**CACCTTGAACTGATAGCACCAA
*sypA*-F53A mutation	4087	ACACGTAGCC**gc**TTTAGATTCATCAGGTATTGGCG
*sypA*-F53A mutation	4088	ATGAATCTAAA**cg**GGCTACGTGTGATAAATCGAT
*sypA*-D22A mutation	4167	GGTGATATGG**ca**GCCATCGGTTGTAGAGATATTC
*sypA*-D22A mutation	4168	CAACCGATGGC**tg**CCATATCACCTTGAACTGATAGC
*sypA*-S56D mutation	4171	CCTTTTTAGAT**gat**TCAGGTATTGGCGCTATTG
*sypA*-S56D mutation	4172	CAATACCTGA**atc**ATCTAAAAAGGCTACGTGTG
*sypA*-S57A mutation	4173	CTTTTTAGATTCA**g**CAGGTATTGGCGCTATTGTT
*sypA*-S57A mutation	4174	GCCAATACCTGcTGAATCTAAAAAGGCTACGTG
*sypA*-D20A, D22A mutations	4245	AGTTCAAGGTG**c**TATGG**ca**GCCATCGGTTGTAGAGATATTC
*sypA*-D20A, D22A mutations	4246	CAACCGATGGC**tg**CCATA**g**CACCTTGAACTGATAGCACCAA

^1^Lowercase letters indicate linker sequences, non-native sequences, and/or *sypA* point mutations, the latter of which are also shown in bold-faced type.

Similar methods were used for generating the wild-type *sypA* complement and, subsequently, alleles that expressed SypA variants, all of which were positioned in the *V. fischeri* genome between the genes *yeiR* and *glmS* as described previously [38]. The *sypA* complement was generated by using PCR to amplify three pieces, an “upstream” region containing a portion of *yeiR* and Erm^R^ (amplified from pKV502), the *sypA* gene and its associated promoter (amplified from ES114) and a “downstream” region containing a portion of the *glmS* gene (amplified from pKV503). The fragments were fused together using PCR SOEing [43] and introduced into the intergenic space between *yeiR* and *glmS* in wild-type strain ES114 carrying *tfoX*-overexpressing plasmid pJJC4 [40]. Genomic DNA isolated from the resulting strain was used 1) as a template for PCR reactions for strain verification and sequence analysis, 2) as a donor for introducing the allele into a *sypA* strain, and 3) as a template in PCR reactions for generating point mutations in *sypA* as follows. Reverse and forward mutagenic primers, respectively, were combined with a forward primer in the Erm^R^ cassette and a reverse primer in *glmS*. The fused DNA fragments were used to transform an intermediate strain (pJJC4-containing KV8232 [38]) that facilitates homologous recombination in that region. Following sequencing to verify the presence of point mutations, genomic DNA was used to introduce *sypA* alleles into the pJJC4-containing Δ*sypA* mutant with selection for the linked Erm^R^ cassette.

### Biofilm spot assay

To test the ability of SypA variants to induce biofilm formation despite the presence of active SypE, the *sypG* overexpression plasmid pEAH73 [25] was introduced into the strains by conjugation and selection for Cm^R^. These strains were grown overnight in LBS containing Cm, then subcultured into the same medium at 28°C. After growth to mid-log phase, cultures were normalized to an OD_600_ of 0.2 and an aliquot of 10 μL was spotted onto LBS Cm plates. After the spots dried, the plates were inverted and incubated for 48 h at 28°C. After incubation, images of the spots were captured using a Zeiss Stemi 2000-C dissecting microscope.

To test the activity of SypA variants, we evaluated their ability to complement a Δ*sypA* mutant under conditions in which SypE is non-inhibitory, *i.e*., during overexpression of *rscS* from plasmid pKG11 [23]. When overproduced under these LBS conditions, RscS inhibits the kinase activity of SypE, permitting SypA to be unphosphorylated and thus active [26]. The pKG11-containing strains were grown and analyzed as described above for SypG overproduction, with the exception that the plates were incubated at 24°C.

## Results

### Computational prediction of SypA

**Figure 2.**
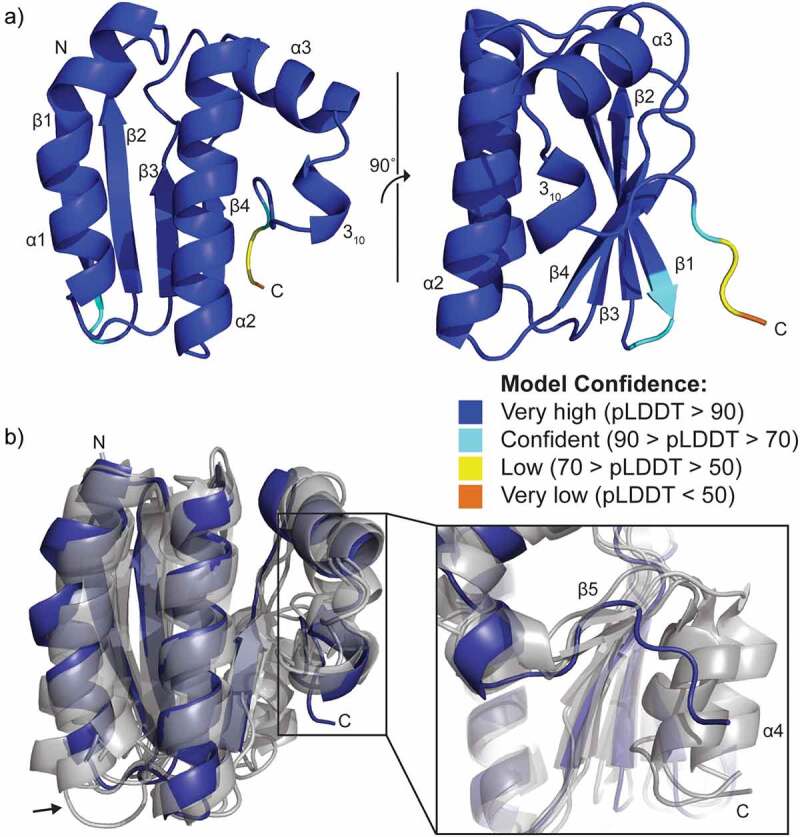
Computational model of SypA and structural alignment to canonical single-domain STAS proteins. a) AlphaFold prediction of *V. fischeri* SypA (Uniprot ID Q5DYQ6). Residues are colored based on the AlphaFold prediction confidence scores (pLDDT) which range from 0 to 100. b) Structural alignment of AlphaFold SypA prediction (blue) with SpoIIAA and RsbV structures (grey; PDB ID 1AUZ, 1THN, 1H4X, and 6M36). The black arrow denotes the structural variability in the α1-β3 loop. The box highlights the C-termini.

A computational prediction of *V. fischeri* SypA (Uniprot ID Q5DYQ6) was generated by AlphaFold2 [44,45] (Figure 2a). Most of the structure, 97 of 105 residues, have a very high confidence score (pLDDT>90). A low confidence score is only present for the last three residues of the C-terminus. This suggests that we can approach the predicted backbone conformation of the SypA model with a reasonable degree of certainty. RoseTTAFold [31] produced a SypA prediction in close agreement to the AlphaFold prediction with a Cα root-mean-square deviation (RMSD) of 0.395 Å. In the absence of an experimentally determined structure of SypA, the close agreement of these two computational predictions indicates the SypA model is as accurate as possible given the current limitations of structure prediction.

**Figure 3.**
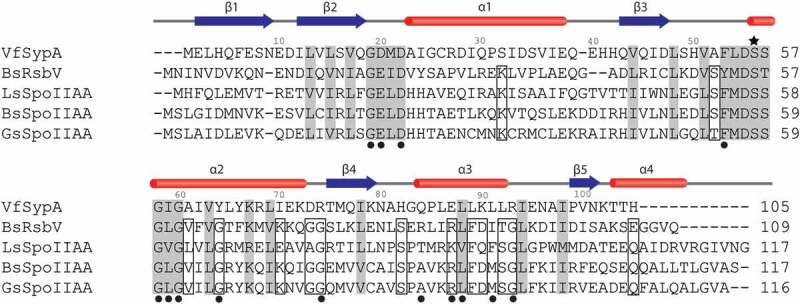
Sequence alignment of SypA, RsbV, and SpoIIAA. Alignments are based on protein secondary structures. The conserved phosphorylation site is denoted with a star. Residues labeled with a closed circle are sites of interest from studies of *B. subtilis* SpoIIAA and RsbV. Shading denotes sequence similarity across all species. Open boxes denote residues that are similar in SpoIIAA and RsbV but from which SypA deviates. Alpha helices and beta sheet strands are indicated above the alignments by red bars and blue arrows, respectively. VfSypA = *V. fischeri* SypA (Uniprot ID Q5DYQ6), BsRsbV = *B. subtilis* RsbV (Uniprot ID P17903), LsSpoIIAA = *L. sphaericus* SpoIIAA (Uniprot ID O32723), BsSpoIIAA = *B. subtilis* SpoIIAA (Uniprot ID P10727), GsSpoIIAA = *G. stearothermophilus* SpoIIAA (Uniprot ID O32726).

The predicted SypA secondary structure closely matches other single-domain STAS protein structures (Figure 2b). Superimposing 19 experimentally determined structures of single-domain STAS proteins with the SypA AlphaFold2 prediction resulted in an average Cα RMSD of 0.825 ± 0.44 Å (data not shown). The overall STAS domain fold is composed of a central five-stranded β-sheet (β1–β5). One side of the β-sheet is overlaid by three α-helices (α1–α3). A short 3_10_-helix extends off helix α3. The opposite face of the β-sheet is partially covered by helix α4 (Figure 2b). The phosphorylation site resides in the β3-α2 loop near the N-terminus of helix α2 (Figure 3).

Since SpoIIAA and RsbV are biochemically and structurally characterized for their role as anti-sigma factor antagonists, we focused on these proteins for comparison to SypA. The SypA prediction shares nearly identical secondary structural similarity to SpoIIAA and RsbV with only three prominent deviations: the β1–β2 loop, the α1–β3 loop, and the C-terminus. Variability in the β1-β2 and α1-β3 loops are the result of protein-specific insertions and deletions in this region. Alterations to the length of the α1-β3 loop impacts the C-terminal angle of helix α1. Both loops have very low sequence similarity across single-domain STAS proteins, suggesting that these minor variations in loop conformation are not significant to the fold or function of the STAS protein (Figure 3).

More notably, the prediction of SypA lacks a well-formed β5 and no α4 (Figure 2b). This is likely due to the truncation of the SypA sequence relative to many other single-domain STAS proteins (Figure 3). Based on the *B. subtilis* SpoIIAA NMR structure, the α4 helix is a well-defined component of other single-domain STAS proteins [8]. The lack of ordered structure at the C-terminus of the SypA prediction suggests two distinctly different conclusions for this region of SypA. First, the C-terminal tail may simply be unimportant for SypA function. Alternatively, it is possible that SypA requires this region to be flexible to adopt a range of potentially function-dependent conformations.

### Structural alignment

Given the minor putative structural differences, we hypothesized that the SypA amino acid sequence maintains key features of a STAS protein while also containing distinct elements that will distinguish SypA from canonical single-domain STAS proteins. To test this hypothesis, we performed a structure-based multiple sequence alignment between SypA, SpoIIAA, and RsbV (Figure 3). SypA shares 24% sequence identity and 58% similarity with *B. subtilis* SpoIIAA, and 19% identity and 55% similarity with *B. subtilis* RsbV. Sequence similarity is concentrated around the Ser56 phosphorylation site, predominately at the N-terminus of helix α2 and the β2-α1 loop. Residues in this region are either directly involved in stabilizing the phosphorylation event or in facilitating binding of the STAS proteins and their anti-sigma factor [9,11,46,47].

The protein-protein binding interactions between SpoIIAA and RsbV with their respective kinases have been well characterized, and residues of interest have been identified (Figure 3) [8–12,46,47]. While SypA maintains many of these residues, there are notable deviations, especially around helix α3. Specifically, two glycine residues conserved in SpoIIAA and RsbV are exchanged in SypA for arginine at positions 74 and 93. Arg74 is located in the α2-β4 loop, on the opposite end of helix α2 from Ser56. Based on the AlphaFold2 prediction, Arg74 may form a hydrogen bond with the backbone of residues 39 and 40 in the α1-β3 loop. Similar hydrogen bonding interactions are not observed in the *B. subtilis* SpoIIAA and RsbV structures. Arg 93 resides just beyond the C-terminus of helix α3 and is surface exposed. Additionally, SypA Tyr64 is a conserved glycine in SpoIIAA and RsbV. Tyr64 is positioned in the middle of α2, just below the phosphorylation site. Along with being a glycine to tyrosine substitution, Tyr64 is solvent exposed. We consider the potential implications of these sequence deviations in the Discussion section below.

Other minor variations are present between the SypA and SpoIIAA and RsbV sequences. Some of these differences simply involve the exchange of positively and negatively charged residues (Glu87, Lys90). In some instances, a charged residue in the canonical sequences is exchanged for a hydrophobic or polar residue (Ser32, Ile70). The differences in sequence at the SypA-SypE binding interface are likely due to protein-binding specificity. Overall, based on sequence alignment, there is no clear indication for the functional difference between SypA and the canonical single-domain STAS proteins.

### Putative SypA-SypE binding interface

Since both the sequence and predicted structure of SypA are consistent with canonical single-domain STAS proteins, we hypothesized that we should observe similar features between the SypA-SypE binding interface and those of SpoIIAA-SpoIIAB and RsbV-RsbW. To explore this potential, a prediction of SypA bound to SypE was generated using ColabFold [32]. ColabFold combines AlphaFold2-multimer [48] structure prediction with MMseqs2 [49] homology search allowing for accelerated prediction of multimeric structures. The presence of a REC domain suggests that SypE forms a dimer. Therefore, we set up the ColabFold run to utilize two subunits of SypE in conjunction with one subunit of SypA. The top-ranked ColabFold output prediction was validated with MolProbity [33] resulting in a MolProbity score of 2.67, 1.08% Ramachandran outliers, and clashscore of 51.64. Global energy optimization through ICM-Pro (Molsoft) significantly improved the prediction geometry and contacts with a MolProbity score of 1.14, 0.47% Ramachandran outliers, and clashscore of 1.54.

**Figure 4.**
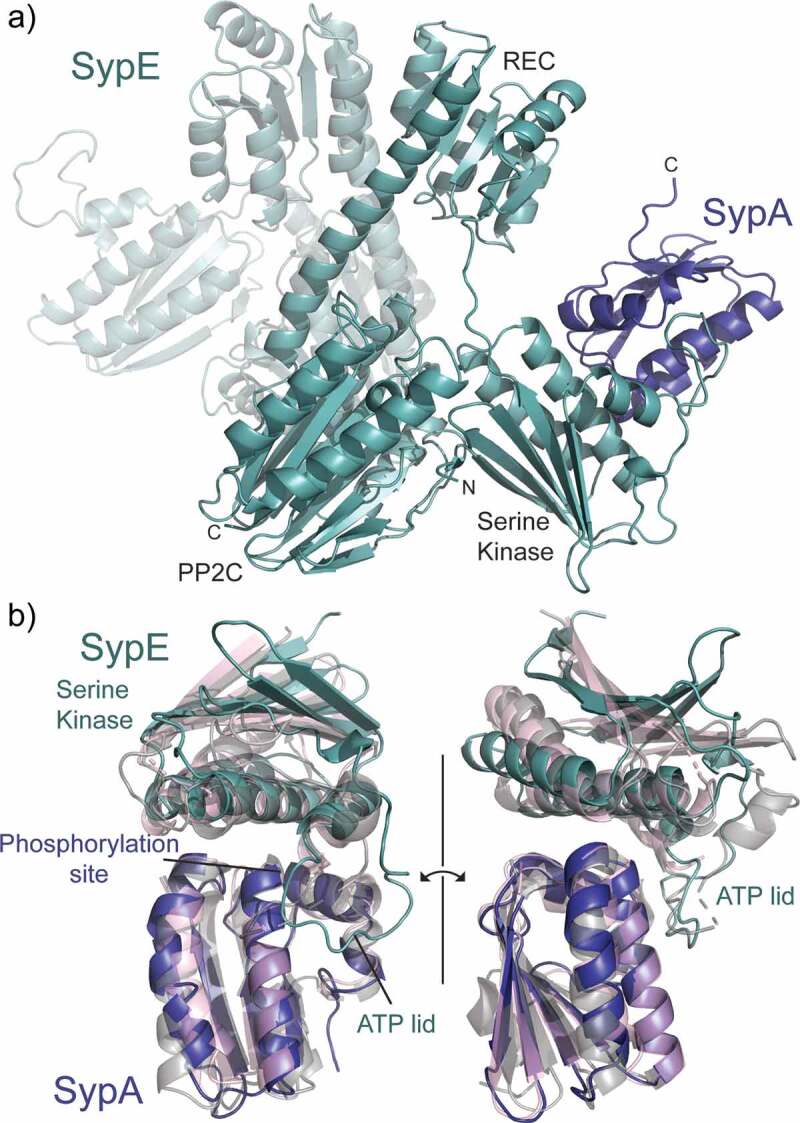
Computational model of SypA-SypE complex. a) Optimized ColabFold prediction of SypA (blue) bound to a SypE dimer (green). The serine kinase REC, and PP2C domains of SypE are labeled. b) Closeup view of SypA bound to the serine kinase domain of SypE. The SypA-SypE prediction is overlaid with the structure of SpoIIAA-SpoIIAB (grey, PDB ID 1TH8) and RsbV-RsbW (pink, PDB ID 6M36). The phosphorylation site of the STAS protein and ATP lid of the serine kinases are labeled.

The resulting computational model predicted a SypE dimer forming through the dimerization interface of the REC domain (Figure 4a). SypA is bound to the SypE-SK domain in a logical position relative to the serine kinase nucleotide binding pocket and in agreement with co-crystal structures of SpoIIAA-SpoIIAB and RsbV-RsbW [9,46] (Figure 4b). Protein-protein interface analysis was carried out using the PISA program [50]. The AlphaFold prediction suggests a SypA-SypE interaction composed of 31 contact residues from SypA and 28 contact residues from SypE. The resulting buried surface area is 1,035 Å^2^. Superimposing SypA-SypE with SpoIIAA-SpoIIAB (PDB ID 1TH8) and RsbV-RsbW (PDB ID 6M36) results in a Cα alignment RMSD of 1.128 Å and 1.056 Å, respectively. The largest structural deviation occurs in the “ATP lid” region of the serine kinase (Figure 4b). This region is unmodeled in the RsbV-RsbW structure and takes on a slightly different conformation in the SpoIIAA-SpoIIAB structure than in the SypA-SypE prediction. The ATP lid is a flexible loop [51] and, therefore, is expected to adopt a range of conformations.

**Figure 5.**
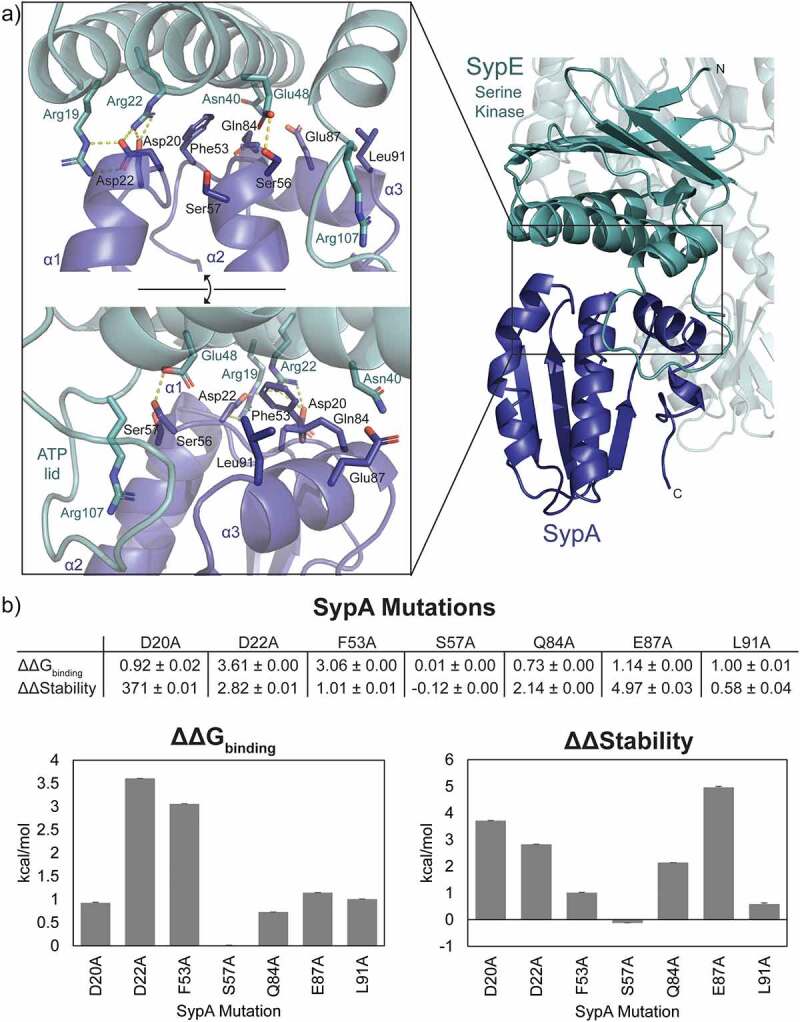
SypA-Sype binding interaction and simulated mutagenesis. a) Binding interface of the optimized ColabFold prediction with SypA (blue) bound to the SypE serine kinase domain (green). SypA residues are labeled in black and SypE residues are labeled in green. Yellow dash lines denote potential hydrogen bonds. b) Simulated mutagenesis data for ΔΔG_binding_ and ΔΔStability for the SypA-SypE binding interaction with data represented in graphical form below. Data are the mean ± standard error in kcal/mol each calculated from 10 simulations.

A closer look at the SypA-SypE interface reveals further similarities to canonical single-domain STAS proteins. A hydrogen bonding network is predicted between SypA Asp20 and SypE Arg 22, SypA Asp22 and SypE Arg19 and Arg22, and SypA Ser56 H and SypE Glu48 (Figure 5). The SpoIIAA and RsbV equivalents of SypA residues Asp20 and Asp22 are known to facilitate anti-sigma factor binding [9,46] and Ser56 is the site of phosphorylation. Additionally, the conserved SypA Phe53 provides hydrophobic contacts at the core of the binding interface.

Residues along SypA helix α3 likely further facilitate the SypA-SypE interaction, but data from the computational model are inconclusive in this region. SypA Glu87 and/or Gln84 may H-bond to SypE Asn40, and SypA Leu91 likely provides hydrophobic contacts to the “ATP lid” of SypE. Similarly, SypA Ser57 should stabilize SypE Arg107 based on the co-crystal structure of *B. subtilis* SpoIIAA and SpoIIAB [9]. The SypE Arg107 counterpart in SpoIIAB interacts with the ATP gamma-phosphate and stabilizes the phosphorylation transition state. The computational model shows no indication for a SypA Ser57 to SypE Arg107 interaction (Figure 5a). This may be due to the absence of ATP in the model. Experimentally derived structures are needed to fully assess the SypA-SypE interaction. Additionally, the presence of a nucleotide bound to SypE may alter which residues facilitate the protein-protein interaction.

### Simulated disruption of the SypA-SypE binding interface

SypA residues Asp20, Asp22, Phe53, Gln85, Glu87, and Leu91 were identified in SpoIIAA and RsbV as being important for the protein-protein interaction between the STAS protein and anti-sigma factor. To explore if these residues could impact SypA-SypE binding, we performed simulated mutagenesis on the optimized ColabFold prediction using ICM-Pro. Changes in the binding free energy of the protein complex with a single residue mutation were calculated using the equation $\Delta \Delta G_{bind}=\Delta G_{binding}^{mutant}-\Delta G_{binding}^{wild\;type}$, where $\Delta G_{binding}=\left(E_{internal}^{complex}-E_{internal}^{parts}\right)+\left(E_{solvent}^{complex}-E_{solvent}^{parts}\right)$ with E^complex^ representing the energy of the protein complex and E^parts^ representing the sum of energies for each protein in the complex. A ΔΔG_bind_>0 kcal/mol denotes reduced binding free energy. Changes in protein complex stability upon mutation were also calculated with the equation $\Delta \Delta G=\Delta G^{mutant}-\Delta G^{wild\;type}$, where ΔΔG >0 kcal/mol predicts destabilization of the complex.

Most tested mutations are predicted to reduce the binding energy between SypA and SypE, with D22A and F53A having the greatest impact with ΔΔG_bind_ values of 3.61 kcal/mol and 3.06 kcal/mol, respectively. E87A and D20A mutations are suggested to have the greatest changes in complex stability with ΔΔStability values of 4.91 kcal/mol and 3.71 kcal/mol, respectively (Figure 5b).

As a control, we also ran simulations with S57A which should not affect the SypA-SypE interaction. The S57A mutation likely has little impact on the SypA-SypE binding interaction. The ΔΔG_binding_ value is only 0.01 kcal/mol and there is a small improvement in complex stability with a ΔΔStability value of −0.12 (Figure 5b). Since Ser57 does not coordinate with Arg107 in our model like it does in the SpoIIAA-SpoIIAB structure [9], it is probable that the simulations could not detect any significant changes in binding energy or complex stability.

Overall, the simulated mutagenesis data suggest that residues that have been found to be important for canonical STAS and anti-sigma factor protein-protein interactions are also likely to be significant in the SypA-SypE interaction.

### Impact of putative SypA-SypE interaction residues on biofilm formation

To begin to assess the modeling predictions described above, we generated and expressed SypA variants with alanine substitutions for residues predicted to be important to the SypA-SypE interface. Specifically, we generated the following SypA variants: D20A, D22A, S57A, and F53A. In addition, as controls, we generated variants with changes to the site of phosphorylation, Ser56. Past work has shown that a change to the conserved site of phosphorylation that prevents phosphorylation, S56A, permits biofilm formation even when SypE is poised to function as an inhibitor, while the phospho-mimic S56D fails to support biofilm formation [20,27].

**Figure 6.**
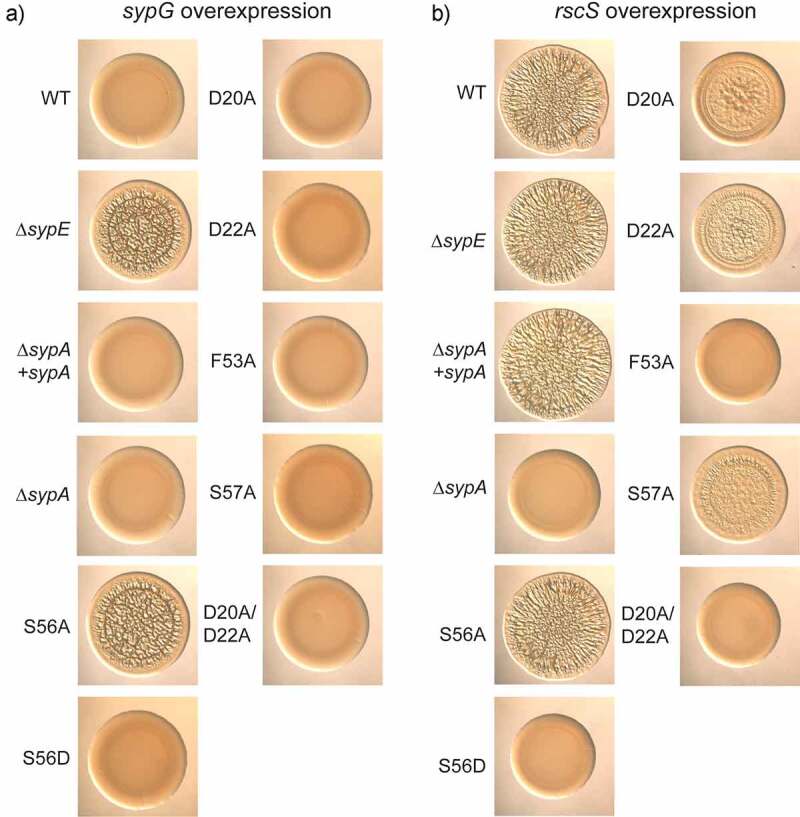
Impact of *sypA* mutations on biofilm formation. Colony morphology at 48 h of strains that carry pEAH73 (*sypG* overexpression) (a) or pKG11 (*rscS* overexpression) (b). The base strains are as described in Table 1: wild type (WT), ES114; Δ*sypE*, KV10149*sypE*; Δ*sypA*, KV10032; Δ*sypA* + *sypA*, KV10163; and Δ*sypA* derivatives that carry the following *sypA* alleles: S56A, KV10247; S56D, KV10331; D20A, KV10248; D22A, KV10334; F53A, KV10249; S57A, KV10332; D20A/D22A, KV10401. Cultures were spotted on LBS plates containing chloramphenicol. Representative images are shown.

We tested biofilm formation under conditions in which SypE functions as an inhibitor of SypA activity, namely multi-copy expression of the response regulator gene *sypG* [27]. When SypG is overproduced, it induces transcription of the genes required for the production of SYP polysaccharide [22], but no SYP-dependent biofilm forms due to the inhibitory activity of SypE on SypA (“WT” in Figure 6a) [27]. This inhibition can be overcome, resulting in wrinkled colony formation, by 1) deletion of *sypE* (Figure 6a) or 2) expression of SypA-S56A (Figure 6A), but not by expression of SypA-S56D (Figure 6a) [27]. When we analyzed wrinkled colony formation by strains carrying the single *sypA* mutant alleles (as the sole copy of *sypA* in the cell), we found that none produced biofilms under these conditions (Figure 6a).

These data suggested that these changes may not be sufficient to disrupt SypA-SypE interactions. As a result, we hypothesized that a greater impact might be observed with multiple changes. We thus generated a variant with two changes, D20A/D22A. However, this strain also did not produce wrinkled colonies under these conditions (Figure 6a).

Given the inability of these variants to promote biofilm formation under SypG overproduction conditions, we wondered if these changes, while potentially disrupting the SypA-SypE interface, also impaired or eliminated SypA activity. To test this possibility, we evaluated the ability of the variants to promote biofilm formation when *rscS* was overexpressed on a multi-copy plasmid. Under these conditions, RscS overproduction prevents SypE-mediated inhibition of SypA [20,26]. The wild-type strain, but not the Δ*sypA* mutant, formed wrinkled colonies under these conditions (Figure 6b). The Δ*sypA* mutant could be fully complemented by the expression of either wild-type SypA or SypA-S56A (Figure 6b). In contrast, SypA variants D20A, D22A, and S57A each exhibited a severely decreased ability to restore full biofilm formation, as the colonies that formed colonies with substantially reduced wrinkled colony architecture with only modest colony stickiness (data not shown) (Figure 6b). Finally, SypA variants F53A and D20A/D22A failed to complement (Figure 6b); these strains formed colonies that were indistinguishable from the negative control. Thus, these single changes and the aspartate double mutant appear to impair or prevent SypA function, a result that potentially accounts for their inability to promote biofilm formation under SypE-inhibitory conditions. We speculate on the possibilities for these results in the Discussion section.

## Discussion

Past work has demonstrated that the single STAS domain protein SypA, along with its partner SypE, functions in a regulatory scheme distinct from other well-known STAS partner-switching systems (Figure 1). To begin to understand how SypA function is distinct from these anti-sigma factor antagonists, we examined its sequence, structure, and protein binding interface. We used a combination of computational modeling and cellular assays to probe the SypA-SypE interaction. Our efforts were guided by the well-studied *B. subtilis* SpoIIAA and RsbV which share sequence homology with SypA.

In lieu of experimentally derived structures, AlphaFold predictions provide us with the means to begin exploring potential structural and functional differences between SypA and SpoIIAA and RsbV. The SypA prediction closely follows the structure of other single-domain STAS proteins, with the greatest similarities located near the protein-protein interface between the STAS protein and serine kinase. This suggests that the mechanisms for facilitating the STAS-kinase interaction are similar between SypA and the canonical STAS proteins SpoIIAA and RspV. The largest structural differences between the STAS proteins lie at the C-terminus of SypA. Since disordered regions are known to facilitate protein-protein interactions [52,53], this unstructured region may facilitate SypA binding to proteins other than SypE. Alternatively, the helix α4 found in other STAS proteins may simply not be necessary for SypA’s role in the cell. Consistent with the latter possibility, SypA with a C-terminal HA-tag retains its functional integrity (Figure 6 and [54]). The mutation of Pro99, located in the short β5 strand adjacent to this region, to alanine resulted in a delay in biofilm formation with no impact of SypA protein expression [54]. This suggests that while the disordered C-terminus may not be important for SypA function, residues in the β5 strand may be. SypA structure determination is necessary to confirm if this region is in fact disordered or is an artifact of the AlphaFold prediction.

Six conserved glycine residues have been identified from the sequence alignments of SpoIIAA- and RsbV-like proteins [11]. SypA lacks three of these glycines and replaces them with Tyr64, Arg74, and Arg93. The substitution of these glycines in SypA appears to be a significant difference between SypA and canonical single-domain STAS proteins, although they do not change the overall STAS domain structure. Tyr64 is of particular interest because it is positioned along helix α2, near the phosphorylation site, and is solvent exposed. Solvent exposed tyrosine residues are favored for mediating protein interactions [55–57]. A SypA Y64A mutation has previously been shown to impact *V. fischeri* biofilm formation [54]. Since SypA likely interacts with proteins other than SypE to carry out its function, Tyr64 may be located in the interface of an unknown protein-protein interaction.

The SypA-SypE binding interaction appears to be nearly identical to that of SpoIIAA and RsbV with their cognate serine kinases [9,46]. The protein binding interfaces share similar features that suggest similar molecular mechanisms. The SpoIIAA and RsbV equivalents to SypA Asp20, Asp22, Phe53, Ser57, Asn84, Glu87, and Leu91 have all been shown to play a role in facilitating the protein binding interaction or are important for phosphorylation [9,11,12,46,47]. Our computational simulations suggest that mutating these residues to alanine should impact the SypA-SypE interaction, except for S57A. Since Ser57 helps stabilize the phosphorylation event [9], the S57A mutation should prevent SypA phosphorylation and, therefore enhance biofilm formation without disrupting the protein binding interaction. Surprisingly, cellular assays did not corroborate the mutagenesis simulations for Asp20, Asp22, Phe53 or Ser57. While similar mutations in SpoIIAA have no impact on protein expression and disrupt the protein-protein interaction with their serine kinase [47], in SypA they failed to increase biofilm formation in the presence of inhibitory SypE. Moreover, the mutants were largely biofilm-defective: the F53A mutant was fully defective for biofilm formation and, while D20A, D22A, and S75A mutants displayed some architecture, they largely lacked the stickiness indicative of SYP polysaccharide production. It is striking that all four mutations predicted to disrupt protein-protein interactions with the inhibitory SypE yielded biofilm defective phenotypes. These results suggest that while SypA shares strong sequence and structural similarities to SpoIIAA and RsbV, how SypA functions with its partners is different from these anti-sigma factor antagonists.

There are three main possible explanations for the failure to regain the biofilm phenotype upon mutation of SypA. The first possible explanation is that the mutations may simply destabilize SypA and interfere with protein expression levels. Given that similar mutations in SpoIIAA do not impact protein expression [47] and that several mutant strains exhibited some level of biofilm production, this does not completely explain our observations.

The second possibility is that by mutating SypA we are impacting its function. This is likely by disrupting another protein-protein interaction. Based on the current Syp regulatory pathway model, we hypothesize that SypA regulates Syp production by interacting with a protein(s) beyond SypE (Figure 1B). While the presence of the surface exposed Tyr64 suggests a secondary non-SypE binding interface, it is possible that another protein involved in SypA function binds in a similar location as SypE. Mutations at this interface that negatively impact interactions with SypE could also negatively impact productive interactions of SypA with another binding partner, in turn impairing biofilm formation. The detrimental effects on biofilm formation of the mutations at the predicted SypA/SypE interface supports this conclusion (Figure 6B).

The third explanation is that the SypA-SypE interaction may be functioning in a manner distinct from the canonical systems. *B. subtilis* serine kinases SpoIIAB and RsbW form at least a dimer, with RsbW potentially forming a tetramer, through salt-bridges along their β1-strand [46]. SypE lacks these residues in its kinase domain. The AlphaFold prediction of dimeric SypE proposes that the dimer forms through the REC domain. The unique architecture of SypE may play a role in how it interacts with and regulates SypA functionality.

Finally, it should be noted that some single-domain STAS proteins are critical components of bacterial stressosomes [58,59]. While SypA shares low sequence similarity to other stressosome STAS proteins, it cannot be ruled out that SypA may play a role in forming a *V. fischeri* stressosome or similar structure. A stressosome has yet to be identified in *V. fischeri*, but one has been found in *V. vulnificus* [60].

## Conclusion

Through comparisons of SypA to the canonical *B. subtilis* single-domain STAS proteins SpoIIAA and RsbV, we found many sequence and structural similarities but also significant differences that may account for SypA’s distinct function. Modeling predicted SypA would bind SypE in a manner similar to that of SpoIIAA and RsbV binding their cognate serine kinases. However, cellular mutational studies demonstrated that SpoIIAA and RsbV mutations known to disrupt these protein-protein interactions did not sufficiently disrupt the SypA-SypE interaction to promote biofilm formation, assuming stability of SypA is maintained. These results align with past studies [20] confirming that SypA does not act as a classical anti-sigma factor antagonist and extend them by demonstrating the critical nature of the residues at the SypE-binding interface. Assuming stability of SypA is maintained, these mutations potentially reveal a SypA surface necessary for binding additional partner(s). Combining computational modeling with wet lab validation via biofilm experiments is thus a powerful approach that has yielded a more comprehensive understanding to the SypA-SypE regulatory pathway. Going forward, given the unusual functionality of SypA and the unique architecture of SypE, structure determination and biochemical analysis of these proteins will help shed light on their cellular functions. In future efforts, we will further explore the SypA-SypE binding interactions, including the SypA-SypE phosphatase domain interaction. We will also seek another SypA binding partner to determine if it binds at the same site as SypE. Ultimately, we anticipate that these studies will help define a new subgroup of STAS domain proteins.

## Data Availability

Data can be found at https://osf.io/euw4c/.
